# Lipopolysaccharide Modifies Glycerol Permeability and Metabolism in 3T3-L1 Adipocytes

**DOI:** 10.3390/ijms18122566

**Published:** 2017-11-29

**Authors:** Jeanne Durendale Chiadak, Patrizia Gena, Françoise Gregoire, Nargis Bolaky, Valérie Delforge, Jason Perret, Giuseppe Calamita, Christine Delporte

**Affiliations:** 1 Laboratory of Pathophysiological and Nutritional Biochemistry, Université Libre de Bruxelles, 1070 Brussels, Belgium; jchiadak@ulb.ac.be (J.D.C.); francoise.gregoire@ulb.ac.be (F.G.); Nargis.Bolaky@ulb.ac.be (N.B.); Valerie.Delforge@ulb.ac.be (V.D.); Jason.Perret@ulb.ac.be (J.P.); 2 Department of Biosciences, Biotechnologies and Biopharmaceutics, University of Bari “*Aldo Moro*”, 70125 Bari, Italy; annapatrizia.gena@uniba.it (P.G.); giuseppe.calamita@uniba.it (G.C.)

**Keywords:** obesity, glycerol, triacylglycerols, aquaglyceroporins, membrane permeability, adipocytes

## Abstract

Aquaglyceroporins—aquaporin membrane channels (AQP) that conduct glycerol and other small neutral solutes in addition to water—play major roles in obesity. In adipocytes, aquaglyceroporins mediate glycerol uptake and release across the plasma membrane, which are two key steps for triacylglycerols (TAGs) synthesis (lipogenesis) and hydrolysis (lipolysis). The aim of this study was to assess both glycerol permeability and metabolism in undifferentiated 3T3-L1 cells (UDCs) as well as in untreated (CTL-DCs) versus lipopolysaccharide (LPS-DCs)-treated differentiated 3T3-L1 adipocytes. Glycerol release, TAGs content and whole membrane glycerol permeability were significantly increased in DCs as compared to UDCs. Moreover, in DCs, LPS treatment significantly increased TAGs content and decreased glycerol permeability. In addition, a significant reduction in whole membrane glycerol permeability was observed in LPS-DCs as compared to CTL-DCs. The relative contributions of AQP3, AQP7 and AQP9 (facilitated diffusion), as well as that of the phospholipid bilayer (simple diffusion), to the whole membrane glycerol permeability, were estimated biophysically in UDCs, CTL-DCs and LPS-DCs, using selective AQP inhibitors. Further studies will be required to determine if modifications in either subcellular localization and/or activity of aquaglyceroporins could account for the data herein. Nevertheless, our findings provide novel insights in understanding the LPS-induced adipocyte hypertrophy that accompanies obesity.

## 1. Introduction

Obesity is characterized by increased adipose tissue mass resulting from both an increase in size of individual adipocytes and the generation of new adipocytes [1]. Adipocytes are made up of more than 95% triacylglycerols (TAGs) that are hydrolyzed to fatty acids and glycerol during lipolysis [2]. Glycerol plays a major role in energy homeostasis, as it represents the carbon backbone of TAGs and is the major substrate for hepatic gluconeogenesis during fasting [3,4,5]. Circulating plasma glycerol results from its release from adipocyte TAGs during lipolysis and its reabsorption by kidney proximal tubules [5,6]. Adipocytes play a central role in whole-body energy homeostasis as they supply energy during starvation. However, over-nutrition and lack of exercise result in over-accumulation of fat that can ultimately lead to obesity [7].

Aquaglyceroporins have emerged as key players in obesity and development of insulin resistance [3,8,9,10]. The main function of aquaglyceroporins in adipocytes is to mediate both glycerol uptake and release, fundamental steps for TAGs biosynthesis (lipogenesis) and hydrolysis (lipolysis), respectively [8]. Aquaglyceroporins represent a subset of the aquaporin (AQPs) family of integral membrane channel proteins [11,12]. To date, 13 mammalian AQPs have been identified: AQP0 to AQP12 [11,12]. AQPs are subdivided into: (*i*) classical AQPs, only permeable to water (AQP0, AQP1, AQP2, AQP4, AQP5, AQP6, AQP8) [11,12]; (*ii*) aquaglyceroporins, permeable to glycerol and other small neutral solutes in addition to water (AQP3, AQP7, AQP9, AQP10) [13,14]; (*iii*) non-classical AQPs, presenting different structural features in the conserved motifs and of debated permeability (AQP11, AQP12) [15]. However, recently, AQP11, though still classified as a non-classical AQP, has been shown to be permeable to glycerol as well [16].

Adipocytes express several aquaglyceroporins that play a role in adipose tissue metabolism and obesity [17,18,19]. Both AQP7 mRNA and protein expression increase with adipocytes differentiation of 3T3-L1 cells [20,21,22]. Glycerol released into the media also increases in parallel with AQP7 mRNA levels in differentiating 3T3-L1 adipocytes [20]. Progressive TAGs accumulation in adipocytes of *Aqp7*-depleted mice resulted in reduced plasma membrane glycerol permeability and subsequent increase of intracellular glycerol concentration, which activates glycerol kinase and increases glycerol-3-phosphate concentration and hence TAGs biosynthesis [23,24]. Under fasting conditions, *Aqp7* knockout (KO) mice exhibit lower plasma and portal glycerol concentrations than wild type (WT) mice [25]. In addition, while no difference in body weight could be observed at a young age between WT and *Aqp7* KO mice, *Aqp7* KO mice became obese after 12 weeks of age [23]. Indeed, adipose tissue weight of *Aqp7* KO mice was significantly higher compared to WT mice at 20 weeks of age. Histological analysis showed an increase in hypertrophic adipocytes in the epididymal white adipose tissue of *Aqp7* KO mice. Moreover, *Aqp7* KO mice exhibited whole body insulin resistance associated with obesity [23]. However, glycerol secretion could not be completely abolished in AQP7-deficient adipocytes [23,25]. While AQP7 was considered to be the sole aquaglyceroporin expressed in adipose tissue [26,27]—expression of AQP3, AQP9 and AQP10 and AQP3 and AQP9 has since been detected in human adipose tissue [28,29,30] and in 3T3-L1 adipocytes [31], respectively. Moreover, AQP3 mRNA levels are higher in 3T3-L1 adipocytes as compared to preadipocytes, while AQP9 mRNA levels are similar [31]. In addition, obesity has been associated with increased AQP3 and AQP9 expression and decreased AQP7 expression in human subcutaneous adipose tissue [28,29]. AQP11, shown to be permeable to glycerol, is also expressed in human adipocytes [16] and mouse 3T3-L1 adipocytes [31]. *Aqp3* KO mice undergo nephrogenic diabetes insipidus [32], *Aqp9* KO can develop diabetes [33] and *Aqp11* KO mice developed polycystic kidneys [34]. Though AQP10 is expressed and could play a role in adipocyte metabolism in human [30], *Aqp10* is a pseudogene in mice [35]. Therefore, it is necessary to be aware and appreciate species differences between mice and human in terms of expression and regulation of aquaglyceroporins [13,18].

Aquaglyceroporins display different subcellular localization in murine 3T3-L1 adipocytes. Indeed, AQP3 is predominantly localized at the plasma membrane and within the intracellular compartment while AQP7 resides predominantly in the intracellular compartments [28]. Both aquaglyceroporins translocate to the plasma membrane upon hormone-induced increase in cyclic adenosine monophosphate (cAMP) while they move from the plasma membrane to the intracellular compartment in response to insulin [28]. However, another study showed that AQP7 internalized upon cAMP stimulation in the mouse white adipose tissue [22]. In addition, AQP9 is constitutively expressed at the plasma membrane and, as in liver [36,37] and appears to undergo translocation upon hormone stimulation [28]. AQP11 has been reported to be primarily located intracellularly in the vicinity of lipid droplets [16].

As obesity is considered an increasing public health issue in developed countries, several studies have been undertaken to identify the pathogenic molecular mechanisms affecting adipocytes. Low level elevations of gut-derived endotoxins (lipopolysaccharide (LPS)) have been shown to play an important role in obesity [38]. Moreover, previous studies have shown that LPS, mimicking inflammation occurring during obesity, can affect the expression of cytokines such as monocyte chemotactic protein-1 [39] and the expression of several aquaglyceroporins [31] in adipocytes. Indeed, LPS decreased AQP7 and AQP11 mRNA levels and conversely increased AQP3 mRNA levels in adipocytes, resulting from the Toll-like receptor 4 (TLR4)-induced activation of the JNK and/or NFκB pathways [31].

Recent data suggest that the increased adipocyte plasma membrane glycerol fluxes may be part of the anti-adipogenic response to conjugated linoleic acid treatments in 3T3-L1 murine differentiated adipocytes [40]. However, it is currently unknown if LPS affects glycerol permeability in adipocytes. The aim of this study was to assess the modification of both glycerol permeability and metabolism in differentiated 3T3-L1 adipocytes exposed to LPS and to estimate the contribution of each one of the investigated aquaglyceroporins to the cellular glycerol permeability.

## 2. Results

### 2.1. Glycerol Release and Triacylglycerols (TAGs) Content in Undifferentiated 3T3-L1 Cells (UDCs), Untreated 3T3-L1 Cells Differentiated into Adipocytes (CTL-DCs) and Lipopolysaccharide-treated 3T3-L1 Cells Differentiated into Adipocytes (LPS-DCs)

**Figure 1:**
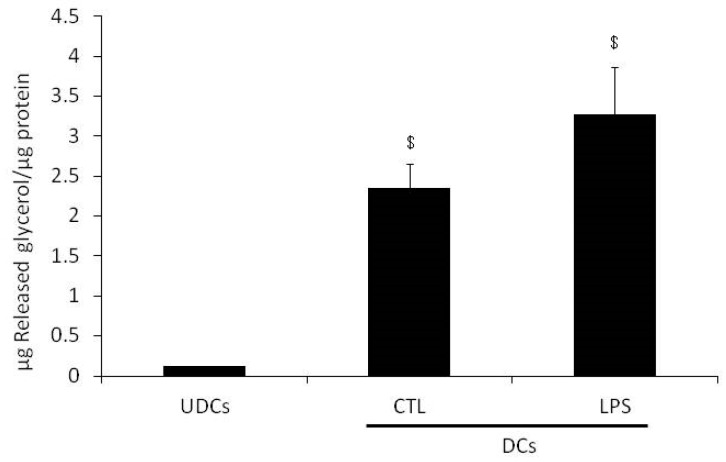
Glycerol release from undifferentiated 3T3-L1 cells (UDCs) and from 3T3-L1 cells differentiated into adipocytes (DCs), untreated (CTL) or treated with lipopolysaccharide (LPS). DCs were treated for 4 h with water (CTL) or 1 μg LPS/mL (LPS). Glycerol release was determined in the culture media as described in Materials and Methods. The results are expressed as μg of released glycerol per μg of protein and are the mean ± S.E.M. of 3 independent experiments performed in triplicate. Data were analyzed using repeated measures One-way ANOVA and Bonferroni post-hoc *t*-test, ^$^ *p* < 0.05 vs. UDCs.

**Figure 2:**
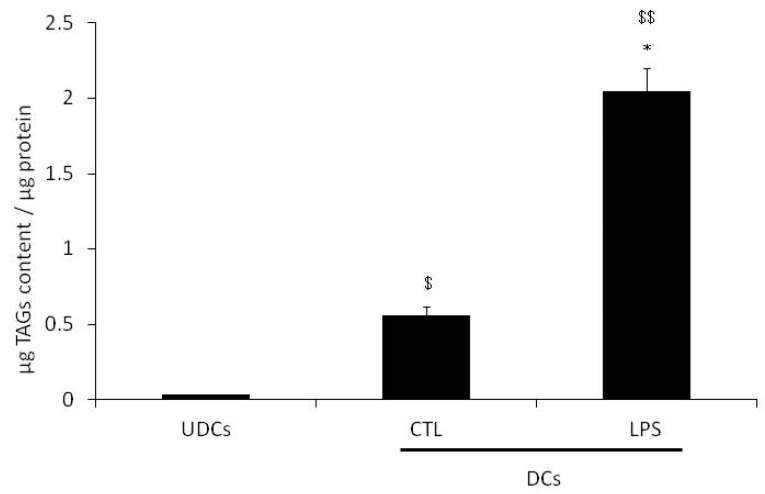
Triacylglycerols (TAGs) content in undifferentiated 3T3-L1 cells (UDCs) and in 3T3-L1 cells differentiated into adipocytes (DCs), untreated (CTL) or treated with lipopolysaccharide (LPS). DCs were treated for 4 h in media supplemented with water (CTL) or 1 μg LPS/mL (LPS). TAGs content was determined following total lipid extraction as described in Materials and Methods. The results are expressed as μg of TAGs content per μg of protein and are the mean ± S.E.M. of 3 independent experiments performed in triplicate. Data were analyzed using repeated measures One-way Analysis of Variance (ANOVA), ^$^ *p* < 0.05 and ^$$^ *p* < 0.01 vs. UDCs, * *p* < 0.05 vs. CTL.

Glycerol release and TAGs content were determined in undifferentiated cells (UDCs) and cells differentiated into adipocytes (DCs) as described in the Materials and Methods section. In CTL-DCs, both released glycerol and TAGs content were significantly increased by 19.6-fold (*p* < 0.05; Figure 1) and 17.5-fold (*p* < 0.05; Figure 2), respectively, as compared to UDCs.

DCs were incubated for 4 h with media supplemented with water or with 1 μg LPS/mL (LPS-DCs) prior to determination glycerol release and TAGs content, as described in Materials and Methods. The released glycerol level from LPS-DCs was significantly higher (27.3-fold) than the level from UDCs. TAGs content from LPS-DCs was significantly increased by 3.7-fold as compared to CTL-DCs (*p* < 0.05) (Figure 2). TAGs content in LPS-DCs was significantly higher (68.3-fold) than that in UDCs.

### 2.2. Biophysical Analysis of UDCs, Untreated DCs and LPS-Treated DCs Membrane Permeability to Glycerol

Adipocyte membrane permeability to glycerol was assessed by a stopped-flow light scattering approach, using plasma membrane vesicles prepared as described in Materials and Methods. Inhibitors of the facilitated membrane transport of glycerol were used to assess the specific contributions of AQP3, AQP7 and AQP9 as well as flow due to simple diffusion through the membrane phospholipids bilayer. Phloretin (0.7 mM, 10 min)—a known inhibitor of the facilitated transport of glycerol [41]—was used to evaluate the relevance of the simple diffusion pathway. HgCl_2_ (0.3 mM, 5 min), was used for its ability to block AQP3 and AQP9 but not AQP7 (amongst mammalian AQPs, HgCl_2_ blocks all AQPs except AQP4 and AQP7) [42]. CuSO_4_ (1 mM, 5 min) was used to inhibit the AQP3-mediated glycerol transport [43]. Light scattering data were expressed as the rate constant *Ki* (s^−1^) of vesicles volume changes following the movement of glycerol across the membrane (see Materials and Methods for details), an index directly reflecting the membrane glycerol permeability.

**Figure 3:**
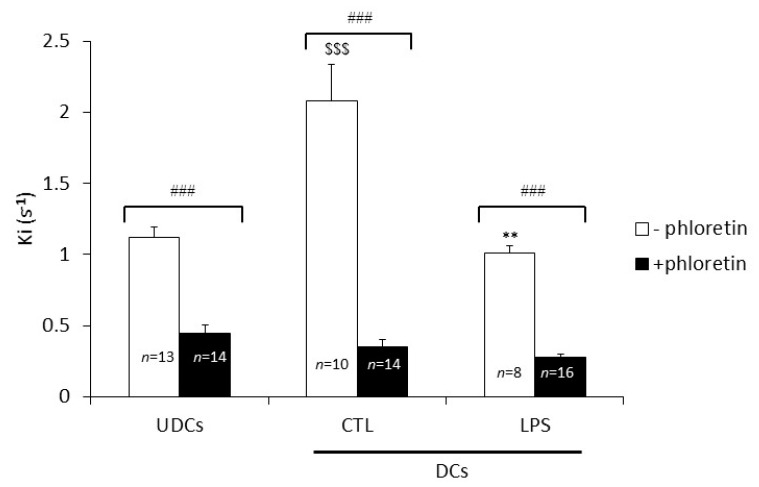
Relative contributions of the facilitated (aquaglyceroporin-mediated) and simple diffusion (phospholipid bilayer) flow through the plasma membrane to the glycerol permeability of UDCs, untreated DCs (CTL) and LPS-treated DCs (LPS). The *Ki* (s^−1^) values, an index reflecting the membrane glycerol permeability, were determined by stopped-flow light scattering analysis of plasma membrane vesicles prepared from UDCs, untreated DCs (CTL-DCs) and LPS-treated DCs (LPS-DCs) in the absence or presence of phloretin (0.7 mM, 10 min). The results are the mean ± S.E.M. of the *Ki* values of 8 to 14 independent vesicle preparations. Data were analyzed using One-way ANOVA and Bonferroni post-hoc *t*-test. ^###^ *p* < 0.001 vs. phloretin; ^$$$^ *p* < 0.001 vs. UDCs; ** *p* < 0.01 vs. CTL.

In absence of inhibitors, the extent of the *Ki* was significantly higher (+85%) in CTL-DCs compared to UDCs (*p* < 0.001), whereas *Ki* was significantly lower (−52%) in LPS-DCs as compared to CTL-DCs (*p* < 0.01) (Figure 3). The glycerol permeability of UDCs, CTL-DCs (CTL) and LPS-DCs was significantly decreased by the aquaglyceroporin inhibitor phloretin as the respective *Ki* values dropped by approximately 60%, 83% and 73%, respectively, following treatment with the inhibitor (*p* < 0.001; Figure 3). Therefore, the relative contribution of the facilitated glycerol pathway, i.e. the one mediated by aquaglyceroporins, is about 60%, 83% and 73% of the whole glycerol permeability of UDCs, CTL-DCs and LPS-DCs, respectively. In the presence of phloretin, the remaining glycerol permeability, representing the simple glycerol diffusion across the lipid bilayer, accounts for about 40%, 17% and 27% of the whole glycerol permeability of UDCs, CTL-DCs and LPS-DCs, respectively.

The relative contributions of AQP7 and AQP3 to the whole plasma membrane glycerol permeability were evaluated with vesicles from UDCs, CTL-DCs and LPS-DCs in the absence or presence of 0.3 mM HgCl_2_, an AQP inhibitor ineffective on AQP7 [42], or 1 mM CuSO_4_, a blocker of the AQP3 permeability [43].

**Figure 4:**
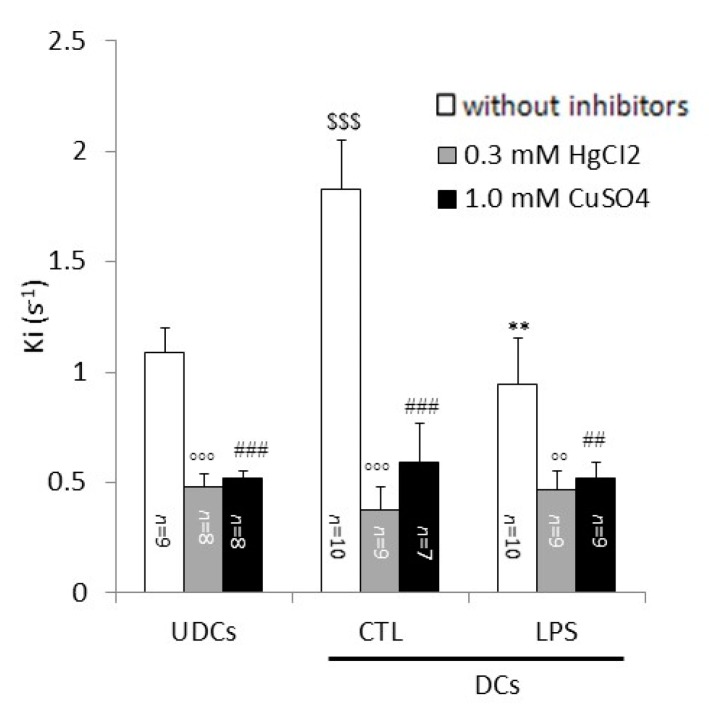
Relative contributions of AQP7 and AQP3 to glycerol permeability in plasma membrane vesicles from UDCs, CTL-DCs and LPS-DCs. *Ki* values were determined by stopped-flow light scattering analysis of plasma membrane vesicles prepared from UDCs, untreated DCs (CTL) and LPS-treated DCs (LPS) in the absence or presence of 0.3 mM HgCl_2_ or 1.0 mM CuSO_4_ as described in Materials and Methods. The results are the mean ± S.E.M. of the *Ki* values from 7 to 12 independent vesicles preparations. Data were analyzed using One-way ANOVA and Bonferroni post-hoc *t*-test. For HgCl_2_: °° *p* < 0.01 and °°° *p*< 0.001 vs. without inhibitors; for CuSO_4_: ^##^ *p* < 0.01 and ^###^ *p* < 0.001 vs. without inhibitors; ^$$$^ *p* < 0.01 vs. UDCs without inhibitors; ** *p* < 0.01 vs. CTL without inhibitors.

Pre-incubation with HgCl_2_ significantly decreased the *Ki* of the UDCs, CTL-DCs and LPS-DCs membrane vesicles by 56%, 80% and 51% (*p* < 0.01), respectively, as compared to the values measured with the vesicles that were not exposed to HgCl_2_ (Figure 4).

Significant inhibition of the glycerol permeability was also seen when the vesicle specimens were treated with CuSO_4_. Following treatment with CuSO_4_, the *Ki* decreased by 52%, 68%, 45% (*p* < 0.01) in UDCs, CTL-DCs and LPS-DCs, respectively, when compared to the absence of the inhibitor (Figure 4).

### 2.3. AQP7, AQP9 and AQP3 Protein Expression in UDCs, CTL-DCs and LPS-DCs

**Figure 5:**
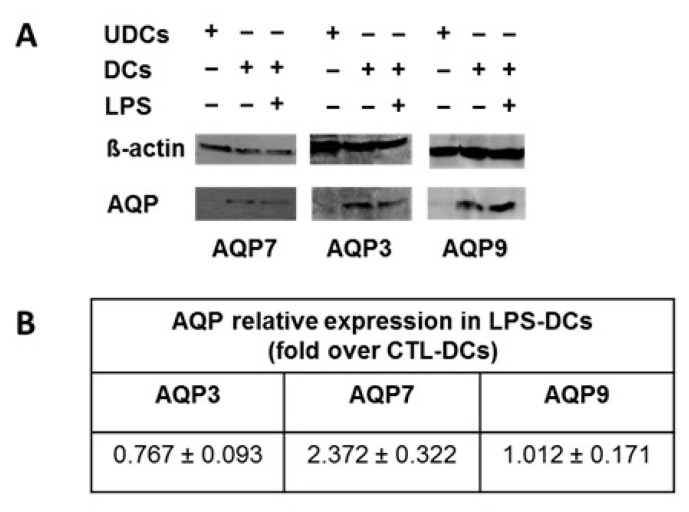
AQP3, AQP7 and AQP9 protein expression in UDCs, CTL-DCs and LPS-DCs. AQP3, AQP7 and AQP9 protein expression levels were determined in UDCs, untreated DCs (CTL-DCs) or LPS-treated DCs (1 μg LPS/mL) by Western blotting analysis. (**A**) AQP7 (30 kDa), AQP3 (30 kDa), AQP9 (30 kDa) and β-actin (42 kDa) were detected using specific antibodies as described in Materials and Methods. Western blot was representative of 3 independent experiments; (**B**) The relative expression of each AQP was determined by densitometry analysis of the Western blots as described in Materials and Methods. Ratio between AQP band volume and β-actin band volume were calculated, the relative expression of each AQP in LPS-DCs is expressed in fold of the ratio calculated for CTL-DCs (set to 1) and is the mean ± standard error of the mean (S.E.M.) of 3 independents experiments. Statistical analysis was performed using the *t*-test for the unique sample.

AQP3, AQP7 and AQP9 protein expression was determined by Western blotting in UDCs, CTL-DCs and LPS-DCs as described in Materials and Methods. No significant AQP7 and AQP3 protein expression and very low AQP9 expression were detected in UDCs. In contrast, AQP3, AQP7 and AQP9 proteins were detected in CTL-DCs (Figure 5). However, the expression of AQP3, AQP7 and AQP9, quantified by densitometry, was not significantly modified in LPS-DCs as compared to CTL-DCs (*p* = 0.129; *p* = 0.051 and *p* = 0.952, respectively; Figure 5).

## 3. Discussion

In the context of obesity and related pathogenic mechanisms involved, LPS has been used to mimic inflammation occurring in the course of the disease. Furthermore, links between aquaglyceroporins expression and obesity have previously been established [20,44,45,46,47]. In addition, it has also been previously shown that LPS can affect the expression of several aquaglyceroporins in 3T3-L1 adipocytes [31]. However, the effects of LPS on glycerol metabolism and/or glycerol permeability in 3T3-L1 adipocytes has not been investigated thus far.

The data from our study show that glycerol released, TAGs content, glycerol permeability and AQP7, AQP9 and AQP3 expression are increased in DCs as compared to UDCs. These data confirm that TAGs accumulation and AQP7 expression increase during adipocyte differentiation occurring during the progression to obesity [20,21,22,27,28]. In addition, LPS treatment of DCs appears to have no considerable effect on release of glycerol and AQP7, AQP9 and AQP3 protein expression, in contrast TAGs content increased and the plasma membrane glycerol permeability decreased.

The contribution of simple diffusion to the whole cell membrane glycerol permeability was estimated by blocking the facilitated diffusion of glycerol with phloretin. The contribution provided by AQP7 was derived by subtracting the *Ki* value obtained in the presence of phloretin from the *Ki* measured with the membrane vesicles treated with HgCl_2_, a compound blocking all adipocyte AQPs except of AQP7. The extent of AQP3-mediated glycerol transport was estimated by subtracting the *Ki* value measured after treatment with CuSO_4_ from the *Ki* measured with vesicles that were not exposed to an inhibitor. The relevance of AQP9 to adipocyte glycerol permeability was estimated by the subtraction of the *Ki* measured after treating the vesicles with HgCl_2_ from the *Ki* measured on vesicles exposed to CuSO_4_. These calculations yielded relative contributions of the simple diffusion pathway, AQP7, AQP3 and AQP9 to the whole glycerol permeability of UDCs to be around 40%, 4%, 52% and 4%, respectively. Based on these data, it can be hypothesized that AQP3 represents the major channel for glycerol permeability in UDCs, fitting in with our previous report showing prevalent localization of AQP3 in the 3T3-L1 adipocyte plasma membrane compared to AQP7 that was found on the other hand to be predominantly localized in the intracellular compartment [28].

The whole adipocyte glycerol permeability of CTL-DCs was significantly higher (+85%) than the permeability measured in UDCs. In addition, based on the calculations described above, the relative contributions of the simple diffusion, AQP7, AQP3 and AQP9 to the whole glycerol permeability in CTL-DCs were estimated to be about 17%, 3%, 68% and 12%, respectively. These data suggest that the increase in the whole glycerol permeability measured in DCs vs. UDCs could result from increased AQP3 and AQP9 expression at the plasma membrane and/or activity. The whole glycerol permeability of LPS-DCs was significantly lower (−52%) than that of CTL-DCs. Furthermore, the relative contributions of simple diffusion, AQP7, AQP3 and AQP9 to the whole glycerol permeability were estimated to be about 27%, 22%, 45% and 6%, respectively. It is therefore reasonable to hypothesize that the decrease in cellular glycerol permeability observed in differentiated adipocytes challenged with LPS (versus CTL-DCs) is due to the reduced expression and/or activity of AQP3 and AQP9 at the plasma membrane compartment. Additional studies designed to specifically knock out a single or several aquaglyceroporin(s) may prove useful in fully assessing the relative contributions of each aquaglyceroporins to the whole cell plasma membrane glycerol permeability.

One of our previous studies showed that the mRNA levels of AQP7 and AQP3 but not AQP9, increased in DCs as compared to UDCs. In addition, LPS had a differential effect in DCs, where it decreased on the one hand AQP7 mRNA levels and increases on the other hand AQP3 mRNA levels [31]. In the present study, both AQP7, AQP9 and AQP3 protein levels increased in DCs as compared to UDCs (in the latter no significant detectable expression of AQP7 and AQP3 and a very low AQP9 expression could be measured), while no significant modification in AQP7, AQP9 and AQP3 protein levels was observed in LPS-DCs as compared to CTL-DCs. In CTL-DCs and LPS-DCs, the apparent discrepancy between the plasma membrane glycerol permeability and AQP7, AQP9 and AQP3 protein expression could be explained by the distinct subcellular localizations of AQP3, AQP9 and AQP7 and/or the additional molecular mechanisms controlling the activity of these aquaglyceroporins. Indeed, aquaglyceroporins display different subcellular distribution in murine 3T3-L1 adipocytes: AQP3 and AQP7 are predominantly localized in the intracellular compartment whereas AQP9 is predominantly localized to the plasma membrane [28]. In addition, upon hormone-induced increase in cAMP, AQP7 and AQP3 translocate from the intracellular compartment to the plasma membrane, while the localization of AQP9 remained unchanged [28]. In human adipocytes, insulin up-regulated AQP7, AQP9 and AQP3 expression and increased intracellular localization of both AQP7 and AQP3 [27]. On the other hand, leptin down-regulated both AQP7 and AQP9 expression and up-regulated AQP3 expression [28]. It was proposed that AQP7 and AQP3 may facilitate glycerol efflux from adipocytes, while AQP9 may facilitate glycerol entry [10,28,48]. However, difference between CuSO_4_-insensitive and HgCl_2_-insensitive isoproterenol-induced glycerol release in human adipocytes appeared to suggest that AQP9 also participated to glycerol efflux during lipolysis [28]. In UDCs, very low amounts of aquaglyceroporins (not detectable or barely detectable by Western blotting analysis) and simple diffusion are likely to account for the measured whole glycerol permeability. Increased AQP9 and AQP3 plasma membrane expression, likely subsequent to their possible modification in subcellular localization and/or activity could account for increased whole glycerol permeability in DCs when compared to UDCs. Despite the lack of modifications observed in AQP7 and AQP3 protein expression levels (using total protein extract) in LPS-DCs as compared to CTL-DCs, the observed decrease in whole glycerol permeability in LPS-DCs versus CTL-DCs may be due to the modification in subcellular localization and/or activity of AQP3 and AQP9. Further studies will be required to follow the subcellular localization of aquaglyceroporins under these conditions.

Studies conducted in *Aqp7* null mice have linked the absence of AQP7 expression to the development of obesity and adipocyte hypertrophy [23,24]. Indeed, *Aqp7* deficiency leads to glycerol retention within adipose tissue, ultimately leading to increased TAGs synthesis and accumulation in adipocytes [23,24]. Moreover, glycerol permeability is reduced in AQP7-lacking adipocytes [24]. Interestingly, in human subcutaneous adipose tissue, obesity is associated with increased AQP3 and AQP9 expression and decreased AQP7 expression [28]. Based on these data and the results from the present study, it is reasonable to hypothesize that low level elevations of systemic gut-derived endotoxins (LPS), known to play an important role in obesity, could lead to decreased glycerol permeability and consequently to increase in TAGs content in adipocytes and thereby contribute to adipocyte hypertrophy. In mice, while AQP7 has been shown to be exclusively expressed in adipose tissue capillaries but not in adipocyte plasma membrane [49], nevertheless AQP7 has also been shown to be expressed in adipocyte plasma membrane in another study [22]. Such distinct findings could account for the differences observed in the susceptibility of *AQP7* knockout mice of distinct genetic background to developing obesity [23,24,50]. However, the role of AQP7 in glycerol transport in adipose tissue capillaries remains to be elucidated using tissue-specific *Aqp7* KO mice. In addition, single or multiple of tissue-specific aquaglyceroporin genes knockout mice will certainly prove to be useful to assess their relative contribution to glycerol metabolism in adipocytes in vivo.

**Figure 6:**
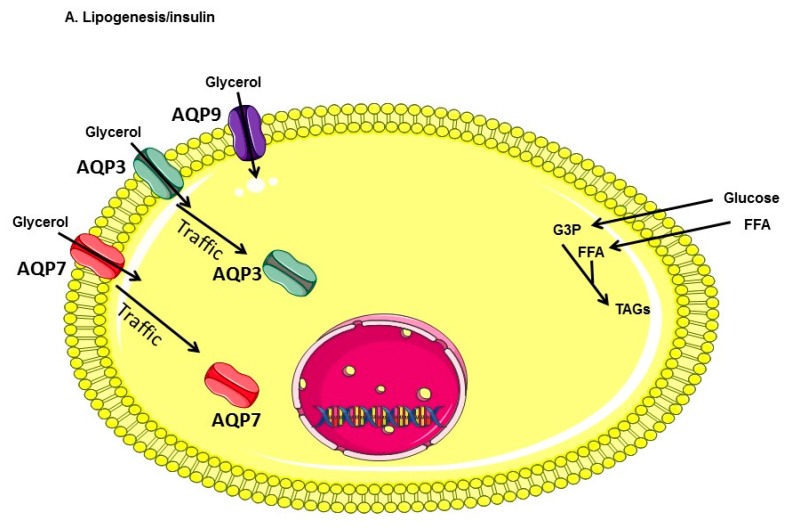

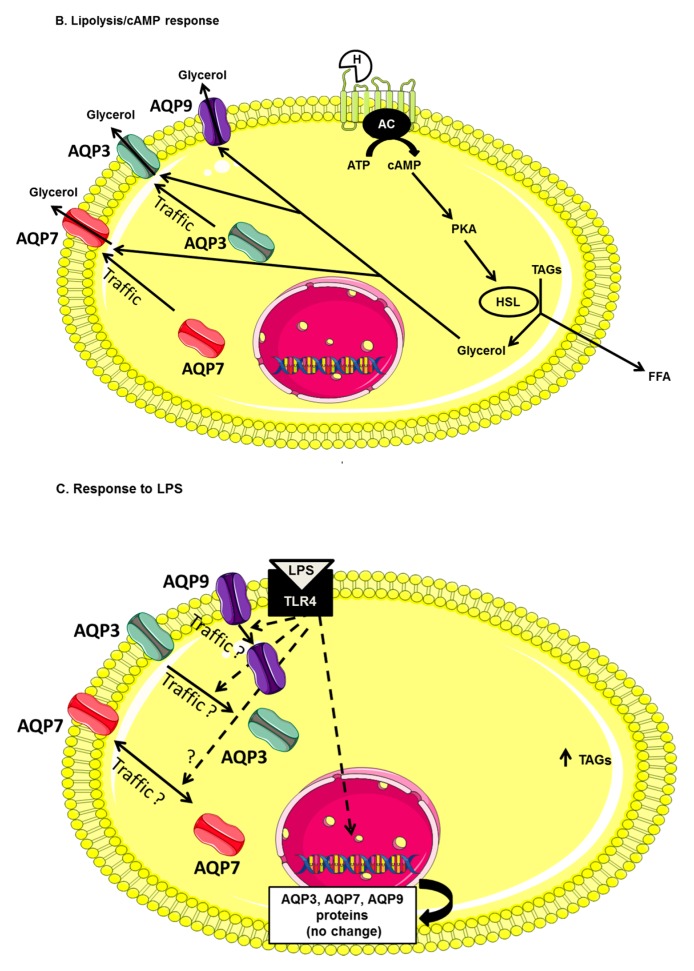
Model for the role of aquaglyceroporins in the regulation of lipogenesis and lipolysis in mouse adipocytes. (**A**) Lipogenesis: insulin is secreted in response to an increase of glycaemia consecutive to feeding. Glucose metabolites, glycerol-3 phosphate (G-3-P) and free fatty acids are used to produce TAGs that will be stored. Both AQP3 and AQP7 are internalized. AQP9 is constitutively expressed at the plasma membrane; (**B**) Lipolysis: upon glucagon secretion in response to fasting or cAMP-mediated response (exercise, catecholamine secretion), TAGs are hydrolyzed to glycerol and free fatty acids, both AQP7 and AQP3 traffic to the plasma membrane enabling increased glycerol efflux. AQP9 may also participate to glycerol efflux during lipolysis. Arrows inside AQP channels represent the direction of glycerol flow; (**C**) Under inflammatory conditions mimicking obesity, LPS is hypothesized to induce AQP3 and AQP9 internalization, thereby reducing plasma membrane glycerol permeability and intracellular TAGs accumulation. Abbreviations: TAGs, triacylglycerols; G-3-P, glycerol-3-phosphate; FFA, free fatty acids; H, catecholamine or glucagon; AC, adenylyl cyclase; ATP, adenosine triphosphate; cAMP, cyclic adenosine monophosphate; PKA, protein kinase A; HSL, hormone sensitive lipase; LPS, lipopolysaccharide; TLR4, Toll-like receptor 4; AQP, aquaporin.

Based on the data obtained in the present work, we propose an updated model of the role of AQP3 and AQP7 in the regulation of lipogenesis and lipolysis in mouse adipocytes (Figure 6). During lipogenesis—upon insulin stimulation—both AQP3 and AQP7 traffic from the plasma membrane to the intracellular compartment (cytoplasm, lipid droplets) and glycerol influx into adipocytes would mainly be ensured by AQP3, despite the additional presence of other aquaglyceroporins (AQP7 and AQP9) (Figure 6A). During lipolysis—upon intracellular cAMP increase—both AQP7 and AQP3 translocate from the intracellular compartment to the plasma membrane and glycerol efflux out of the adipocytes would be ensured essentially by these aquaglyceroporins (Figure 6B). This makes sense form a physiological point of view, as upon lipolysis, the resulting glycerol must exit the cell to be made available to hepatocytes. Based on this model and to account for the decreased plasma membrane glycerol permeability and TAGs accumulation upon LPS treatment of DCs (as compared to CTL-DCs), LPS is hypothesized to induce AQP3 (and possibly AQP9) trafficking from plasma membrane to intracellular compartment, or to modify aquaglyceroporins activity. LPS is thereby likely to contribute to the adipocyte hypertrophy that accompanies obesity. Additional studies will be required to confirm the subcellular localization of aquaglyceroporins, as well as their relative contributions to the glycerol permeability of untreated and LPS-treated adipocytes following single or multiple aquaglyceroporin knockouts of the aquaglyceroporin genes involved.

## 4. Materials and Methods

### 4.1. Reagents

Dulbecco’s modified Eagle’s medium (DMEM, 4.5 g/L glucose), streptomycin/penicillin, fetal bovine serum, horse serum and calf serum were obtained from Invitrogen (Carlsbad, CA, USA). Bovine insulin, 3-isobutyl-1-methylxanthine (IBMX), dexamethasone, LPS, Free Glycerol Reagent, Triglyceride Reagent phloretin, HgCl_2_ and CuSO_4_ were purchased from Sigma (St. Louis, MO, USA). Antibodies directed against AQP3 and β-actin were purchased from EMD Millipore (Burlington, MA, USA). Antibodies directed against AQP7 and AQP9 were purchased from Alomone Labs (Jerusalem, Israel).

### 4.2. Cells Culture

3T3-L1 murine pre-adipocyte cells were kindly provided by Dr. I. Pirson [51] and were grown in DMEM supplemented with 10% calf serum, 200 U/mL penicillin and 200 U/mL streptomycin in an 8% CO_2_, humidified atmosphere at 37 °C until confluence (UDCs). Briefly, adipocyte differentiation was induced 2 days post-confluence by incubating cells for 60 h in Dulbecco’s Mofied Eagle Medium (DMEM) supplemented with 10% fetal bovine serum containing 500 μmol/L IBMX, 0.25 μmol/L dexamethasone and 10 μg/mL insulin, as previously described [21]. The cells were then maintained in the culture medium supplemented with insulin only and this media was changed every 2 days (day 5 and 7) until complete differentiation (monitored by lipid droplet accumulation under the microscope and confirmed by Oil Red Staining) had occurred (day 9). On day 9, DCs were treated for 4 h with media containing water (untreated DCs; CTL-DCs) or 4 h with 1 μg/mL LPS (LPS-treated DCs; LPS-DCs) [31].

### 4.3. Glycerol Release Measurement

Released glycerol (in the culture media) was determined using cells plated in 6 well plates (UDCs, CTL-DCs and LPS-DCs) by using an enzymatic colorimetric method according to the manufacturer’s instructions (SIGMA Free Glycerol Reagent, St. Louis, MO, USA). Released glycerol was expressed as μg glycerol per μg of protein concentration (μg Released glycerol/μg protein).

### 4.4. Triglyceride Measurement

Lipids were extracted from UDCs, CTL-DCs and LPS-DCs plated in 6 well plates as previously described [52]. Briefly, lipids were extracted using 250 μL hexane:isopropanol (3:2) at 4 °C for 15 min and this was repeated after collecting the first organic extract. The second extract was pooled with the first and the solvent was allowed to evaporate. Lipids were then dissolved in 100 μL of isopropanol and TAGs content was measured using an enzymatic colorimetric method according to the manufacturer’s instructions (SIGMA Triglyceride reagent, St. Louis, MO, USA). After lipids extraction, proteins were extracted as previously described [31] and quantified using the BCA protein assay. TAGs content was expressed as μg TAGs per μg of protein concentration (μg TAGs/μg protein).

### 4.5. Western Blot Analysis

Whole cell lysates from DCs, CTL-DCs and LPS-DCs were prepared and submitted to SDS-polyacrylamide gel electrophoresis (SDS-PAGE) in the presence of 5% β-mercaptoethanol using 12% polyacrylamide gels. Cell harvest and whole cell lysates preparation were performed as previously described [21]. Proteins were transferred to polyvinylidene difluoride membranes and immunolabeled using primary antibodies against AQP3, AQP7, AQP9 and β-actin. The bound primary antibodies were detected using secondary anti-mouse or anti-rabbit antibodies (GE Healthcare, Little Chalfont, Buckingham shire, UK) and revealed using an ECL chemiluminescence detection kit (PerkinElmer, Waltham, MA, USA). After exposure to radiographic film, the immunoreactive bands were then scanned and digitized. Densitometry analysis of immunoreactive bands was performed using the Quantity One Software (Bio-Rad, Hercules, CA, USA). Band densities (a measure of “volume”) were estimated by digitalization of the image in pixels, the intensity of which is coded on a scale of grey levels. The measure of the volume represents the sum of all pixel intensities comprising the band and depends on the number of pixels inside the band area and the density of pixels. As AQP expression in UDC was not significant or very low (close to background level), densitometry was only performed for CTL-DCs and LPS-DCs. AQP expression levels in LSP-DCs were expressed as fold over CTL-DCs to avoid bias. Ratio between AQP band volume and β-actin band volume were calculated, the relative expression of each AQP in LPS-DCs is expressed in fold over the ratio calculated for CTL-DC (set to 1) and are the mean ± S.E.M. of 3 independents experiments. Statistical analysis was performed using the *t*-test for each unique sample.

### 4.6. Preparation of 3T3-L1 Plasma Membrane Vesicles

Cells were allowed to grow on 10 cm Petri dishes and treated as described above to obtain UDCs, CTL-DCs and LPS-DCs. After treatment, the Petri dishes were placed on ice, cells were washed 3 times with 5 mL of 20 mM HEPES pH 7.4 containing 250 mM sucrose, 1 mM EDTA and supplemented with EDTA-free Protease Cocktail inhibitor tablets (Roche Diagnostics GmbH, Mannheim, Germany) (1 tablet per 50 mL of buffer), then harvested in 1 mL of buffer per Petri dish, centrifuged at 800× *g* for 15 min at 4 °C and the cell pellets were frozen in liquid nitrogen and stored at −80 °C until they were used to prepare the related plasma membrane vesicles. Cells from 30 Petri dishes per condition were pooled (3 independent experiments, 10 Petri dishes per experiment).

Vesicles of plasma membrane enriched fractions of UDCs, CTL-DCs and LPS-DCs 3T3-L1 adipocytes were prepared as previously described by Sadler and coworkers [53]. Briefly, the frozen cell pellets—described above—were thawed gently and resuspended in ice-cold HEPES/EDTA/sucrose (HES) buffer (250 mM sucrose, 20 mM HEPES pH 7.4, 1 mM EDTA, supplemented with EDTA-Free Protease Cocktail Inhibitor tablets, Roche). Cells were homogenized by passing through a 24 gauge needle ten times and subsequently twice through a 26 gauge needle. Homogenates were centrifuged at 10,000× *g* for 20 min to pellet nuclei, mitochondria and plasma membranes (PM). The resulting pellet was resuspended in HES buffer, layered onto a high sucrose HES cushion (1.12 M sucrose in HES buffer) and centrifuged at 41,000× *g* for 1 h. The layer containing the PM fraction was collected, resuspended in HES buffer and pelleted at 140,000× *g* for 1 h. The resulting pellet enriched in plasma membranes was retained and the protein concentration assayed by the Lowry method. All centrifugations were carried out at 4 °C. Purity of 3T3-L1 plasma membrane enriched fractions was assessed by assaying the activity of the Na^+^-K^+^-ATPase, a plasma membrane marker. Enzymatic assay showed a 11.3-fold enrichment in plasma versus intracellular membranes, a purity comparable to that previously reported [53]. In order to check the quality and evaluate the diameter aliquots of the vesicles, pellets were fixed in a mixture of 3% paraformaldehyde and 1% glutaraldehyde in 0.1 mol/L PBS at pH 7.4 for 2 h at 4 °C. Specimens were postfixed in 1% OsO_4_ in PBS for 1 h at 4 °C. Fixed samples were dehydrated in ethanol and then embedded in Epon (TAAB, Reading, UK). Ultrathin sections were mounted on Cu/Rh mesh grids. Finally, the sections were stained with uranyl acetate and lead citrate and observed using a Zeiss EM 109 electron microscopy (Zeiss, Oberkochen, Germany).

### 4.7. Stopped-Flow Light Scattering Measurement of Glycerol Permeability

Size and quality of plasma membrane-enriched vesicles prepared as described above were determined both by automated size analysis using a N5 Submicron Particle Size Analyzer (Beckman Coulter, Palo Alto, CA, USA) and by morphometric analysis of electron micrographs of the vesicles preparation as previously described [54,55]. The time course of vesicle volume change was followed by way of the changes in the scattered light intensity at 20 °C at a wavelength of 530 nm by using a BioLogic MPS-200 stopped-flow reaction analyzer (BioLogic, Claix, France), having a 1.6 ms dead time and 99% of mixing efficiency in <1 ms. Light scattering experiments were performed as previously reported by submitting the vesicles to a 150 mM inwardly directed gradient of glycerol [36,56]. Data were fitted to a single exponential function and the related rate constant (*Ki*, s^−1^) of the swelling due to the water influx following the entry of glycerol into the vesicle specimen was measured. Under such conditions the *Ki* values represents an index of the glycerol permeability of the analyzed vesicular membranes. In some series of experiments vesicles were resuspended in media deprived of ethylene glycol-bis(β-aminoethyl ether)-*N*,*N*,*N*′,*N*′-tetraacetic acid (EGTA) and ethylenediaminetetraacetic acid (EDTA) prior to incubation with inhibitors such as HgCl_2_ (0.3 mM, 5 min), CuSO_4_ (1 mM, 5 min) and phloretin (0.7 mM, 10 min).

### 4.8. Statistical Analysis

Data are presented as mean ± S.E.M. of n experiments. Group means were compared by repeated measures One-way ANOVA and One way ANOVA and Bonferroni post-hoc *t*-test. Statistical analysis was also performed using a *t*-test for unique samples. Differences were considered statistically significant at *p* < 0.05. All statistical analysis was performed using SPSS 22 (version 22.0.0.0; IBM Corp., Armonk, NY, USA). We thank P. Cullus for helpful guidance in statistical analysis.

## Abbreviations

AQP	Aquaporin
CTL	Control
DCs	3T3-L1 cells differentiated into adipocytes
LPS	Lipopolysaccharide
UDCs	Undifferentiated 3T3-L1 cells
TAGs	Triacylglycerols
cAMP	Cyclic adenosine monophosphate
